# Design and Development of Smoking Cessation Apps Based on Smokers’ and Providers’ Perspectives in China: Survey Study

**DOI:** 10.2196/12200

**Published:** 2019-10-04

**Authors:** Junfang Xu, Jonathan Bricker, Xiaoxing Fu, Chunyan Su, Peicheng Wang, Tengfei Qi, Feng Cheng

**Affiliations:** 1 Center for Health Policy Studies School of Public Health Zhejiang University School of Medicine Hangzhou China; 2 Division of Public Health Sciences Fred Hutchinson Cancer Research Center Seattle, WA United States; 3 Department of Psychology University of Washington Seattle, WA United States; 4 Institute of Anthropology Renmin University of China Beijing China; 5 Media School University of Chinese Academy of Social Sciences Beijing China; 6 Research Center for Public Health School of Medicine Tsinghua University Beijing China; 7 Department of Sociology Tsinghua University Beijing China

**Keywords:** smoking cessation apps, mHealth, design, smartphones, China

## Abstract

**Background:**

Although there are more than 60 smartphone apps for smoking cessation in China, many of them do not include the content and features that health care professionals and smokers prefer—which may make them impractical, unengaging, and ineffective. Therefore, we investigated both health care providers’ and smokers’ preferences for features of future smoking cessation apps.

**Objective:**

This study aimed to investigate Chinese health care providers’ and smokers’ desired features of a smoking cessation app, with the goal of providing design recommendations for app designers and researchers.

**Methods:**

Both Chinese smokers who own smartphones (n=357) and Chinese health care providers (n=224) responded to a survey collecting data on their sociodemographic characteristics and opinions on the importance of 20 smoking cessation app design features studied in previous US research.

**Results:**

Chinese health care providers expressed strong support of smoking cessation apps on a number of attitude indicators (range 153/224, 68.3% to 204/224, 91.1%). They rated nearly all (18/20) features as very or extremely important (range 52.2%-83.4%) and rated nearly all features (17/20) as more important than the smokers did. More than 60% of smokers rated the following 4 features as very or extremely important: allow sharing the process of smoking cessation with family members and friends (216/319, 67.7%), helping smokers track their progress (such as the amount of smoking per day; 213/319, 66.8%), helping with the side effects of medications and nicotine withdrawal symptoms (201/319, 63.0%), and adapting to ongoing needs and interests of smokers (194/319, 60.8%). Contrary to a similar study of US smokers and health care providers, Chinese smokers and providers rated reputation and ability to communicate with family members and friends as important features, whereas Chinese smokers rated privacy and security as less important.

**Conclusions:**

The design of future smoking cessation and health behavior change apps should consider perspectives of both providers and smokers as well as the role of culture.

## Introduction

### Background

The harm caused by tobacco use is one of the most serious public health problems in the world. Although smoking has declined in some countries, it remains prevalent in China [1-3]. The 2015 China Adult Tobacco Survey Report [4] estimated the smoking rate among individuals aged 15 years or older to be 27.7%, which means approximately 316 million smokers live in China. The smoking rate among males is 52.1%, with 60.0% of male smokers aged between 45 and 64 years. Currently, smoking kills more than 1 million people each year, and this number is expected to increase to 2 million if current smoking rates continue [5]. The proportion of the smoking population planning to stop smoking within 12 months has increased from 15.4% in 2010 to 17.6% in 2015 [6]. Correspondingly, many smoking cessation strategies are being attempted, such as nicotine replacement therapy, acupuncture, hypnotherapy, and behavioral interventions [7]. However, traditional quit smoking interventions have had very modest success rates and limited population-level reach [8].

There is a technology that offers a potentially impactful opportunity to change that in China: smartphone apps. In 2013, about 40% of the Chinese population, approximately 556 million people, owned smartphones and that increased to almost 90% of Chinese people (1.3 billion) in 2018 [9]. The ubiquity of smartphone use in China and its projected rise over the next 10 years make smartphones a highly accessible platform for delivering quit smoking interventions to millions of Chinese smokers each year. Therefore, 64 smoking cessation apps have been developed in the mobile app market in 2016, providing new ways to assist smokers in quitting smoking [10].

However, Chinese smoking cessation apps have not proven either popular or effective. One challenge is utilization. For example, research shows that 26% of individuals use their downloaded app only once [11]. The user ratings of smoking cessation apps are also low. Results of current studies in the United States [12,13] and a recent study we conducted in China [10] indicate that these apps still lack most elements that are recommended for quitting smoking. Moreover, studies on smoking cessation apps show that many lack the features that health care professionals and smokers prefer, thereby contributing to their low utilization [14,15].

### Objectives

To date, 1 study, conducted in the United States, has focused on health providers’ and smokers’ desired features of smoking cessation apps [16]. The study showed the features of *free or low cost*, *keeps information private*, *matches individual needs and interests*, and *adapts as one’s needs and interests change* to be very or extremely important [16]. However, the content and features that render smoking cessation apps engaging and appealing to users in the United States may be considerably different in China, given the differences in cultural values and practices between Chinese and Western cultures [17,18]. Therefore, the aim of this study was to investigate Chinese health care providers’ and smokers’ desired features of a cessation app, with the goal of providing design recommendations for app designers and researchers.

## Methods

### Samples

The samples were adult Chinese smokers and health care providers. Regarding the smoker sample, they were volunteers recruited via smoking cessation clinics in Beijing and Web-based advertisements. For those recruited via clinics, all smokers who visited the smoking cessation clinics during May 1, 2016, and June 1, 2016, were invited to participate in our study. In total, 38 eligible smokers with face-to-face interviews were included in our study. For those recruited via Web-based advertisements, we used WeChat (Tencent Inc) and QQ (Tencent Inc), 2 of the most popular multipurpose messaging social media platforms in China. (For the reader’s reference, WeChat was downloaded on 94% of China’s smartphones, with 806 million active users each month in 2016. The number of active accounts in QQ was 808 million per month in 2014 [19,20].) Interested and eligible smokers were invited to provide Web-based feedback on their preferences for the features of smoking cessation apps. The questionnaires used in the Web-based and in-person interviews were the same. Smokers’ inclusion criteria were that they should (1) be aged 18 years or older, (2) own a smartphone, and (3) smoke at least one cigarette every day. The exclusion criteria were that they (1) were aged 18 years or younger, (2) smoked less than daily, (3) could not use apps installed on the smartphone, and (4) had 30% data missing from the questionnaire [21].

Regarding the health care provider sample, they were recruited through convenience sampling and snowball sampling. We started with physicians who worked in pulmonary clinics and specialized in treating nicotine dependence. We then asked them to recommend other related health care providers. In total, 319 smokers and 224 health care providers were included in the data analysis.

### Data Sources and Analysis

We collected data using face-to-face interviews with a questionnaire and a Sojump Web-based questionnaire survey software between May 1, 2016, and August 31, 2016, for smokers and March 25, 2019, and May 5, 2019, for health care providers. Sojump is a secured Web-based survey platform that can administer surveys via QQ or WeChat. The survey collected the following information: (1) demographic characteristics and other basic information (Tables 1-3) and (2) a rating of 20 possible smoking cessation app features first described in a previous study of US smokers and providers [16]. The authors deleted 1 feature (ie, program lets you communicate with your personal doctor or health care team) because the vast majority of people in China are not assigned to personal doctors or health care team members. The attitudes and beliefs about smoking cessation apps from both providers’ and smokers’ perspectives were collected using a 5-anchor rating scale (completely disagree, somewhat disagree, neutral, somewhat agree, and completely agree).

Both health care providers and smokers were asked to rate the importance of each feature using a 4-anchor rating scale (not at all, somewhat, very, and extremely). The features were categorized into 5 domains developed in the previous study of US smokers [16]. The first domain was cost: program is low cost or free. The second domain was reputation: being *research tested*, endorsed by clinical experts, and highly rated by others. The third domain was privacy and security: confidentiality of information, information stored on mobile phone, and information stored in secured *cloud*. The fourth domain was supportive content and user experience: help smokers track their progress, help with the side effects of medications and nicotine withdrawal symptoms, match personal needs and interests, adapt to ongoing needs and interests of smokers, include information about smoking cessation medications, can send out auxiliary or mobility information, include stories about the experiences of quitting smoking from smokers, include videos about quitting smoking, and include games or entertainment. The fifth domain was communication: allow communication between smokers and experts on smoking cessation; allow smokers to communicate with other smokers; allow sharing the process of smoking cessation with family members and friends; and allow smokers to disclose information on microblogs, WeChat, or other social networking sites.

The features were chosen to (1) reflect technology-based strategies for implementing best practice treatment recommendations (eg, addressing use of pharmacotherapy, providing social support, and offering cognitive behavioral–based content), (2) reflect ways to leverage other smartphone capacities to make these programs more engaging (eg, gaming), (3) assess perceived limitations of mobile health (mHealth) tools (eg, security and privacy), or (4) understand other user preferences that may inform future program development (eg, cost and reputation). These features were fully consistent with the US study [16] and thus made it comparable to that study.

### Statistical Analysis

Frequencies and percentages were used to describe the opinions or beliefs about smoking cessation apps and the features. We analyzed whether the importance of the rated features differed significantly among subgroups of the sample using the Mann-Whitney *U* and Kruskal-Wallis tests.

**Table 1:** Demographic characteristics of smokers (N=319).

Variable and category		Statistics
**Gender, n (%)**		
	Male	295 (92.5)
	Female	24 (7.5)
**Age (years), n (%)**		
	≤25	83 (26.0)
	26-35	127 (39.8)
	≥36	109 (34.2)
**Occupation, n (%)**		
	Full time worker	148 (46.4)
	Student	40 (12.5)
	Other	131 (41.1)
**Education level, n (%)**		
	High school or below	46 (14.4)
	Bachelor’s or senior college degree	195 (61.1)
	Master’s degree or above	78 (24.5)
**Nationality, n (%)**		
	Han	283 (88.7)
	Other	36 (11.3)
**Living region, n (%)**		
	Metropolis (ie, Beijing, Shanghai, and Guangzhou)	122 (38.2)
	Provincial capital^a^	81 (25.4)
	Prefecture-level city^b^	68 (21.3)
	County level or other	48 (15.1)
**Living status, n (%)**		
	Living alone	53 (16.6)
	Living with family	212 (66.5)
	Living with roommates	47 (14.7)
	Other	7 (2.2)
**Tried to quit smoking before, n (%)**		
	Yes	187 (58.6)
	No	132 (41.4)
**Used other health-related app, n (%)**		
	Yes	68 (21.3)
	No	251 (88.7)
**Downloaded smoking cessation app before, n (%)**		
	Yes	14 (4.4)
	No	305 (95.6)
Average cigarettes per day, mean (SD)		15 (12)

^a^A provincial capital is the city exercising primary status in a state, province, or an autonomous region, usually as its seat of local government.

^b^A prefecture-level city is an administrative division of China, ranking below a province and above a county in China’s administrative structure.

**Table 2:** Demographic characteristics of health care providers (N=224).

Variable and category		Statistics
**Gender, n (%)**		
	Male	40 (17.9)
	Female	184 (82.1)
**Age (years), n (%)**		
	≤25	31 (13.8)
	26-35	104 (46.4)
	≥36	89 (39.7)
**Occupation, n (%)**		
	Physician	81 (36.2)
	Hotline worker	1 (0.4)
	Health education commissioner	13 (5.8)
	Social worker	16 (7.1)
	Nurse	101 (45.1)
	Others	12 (5.4)
**Education level, n (%)**		
	High school or below	3 (1.3)
	Bachelor’s or senior college degree	161 (71.9)
	Master’s degree or above	60 (26.8)
**Nationality, n (%)**		
	Han	218 (97.3)
	Other	6 (2.7)
**Living region, n (%)**		
	Metropolis (ie, Beijing, Shanghai, and Guangzhou)	123 (54.9)
	Provincial capital	66 (29.5)
	Prefecture-level city	35 (15.6)

**Table 3:** Providers’ attitudes and beliefs about smoking cessation apps.

Items	Completely agree, n (%)	Somewhat agree, n (%)	Neutral, n (%)	Somewhat disagree, n (%)	Completely disagree, n (%)
Many of my clients or patients use mHealth^a^ to manage their health.	67 (29.9)	82 (36.6)	65 (29.0)	7 (3.1)	3 (1.3)
mHealth apps hold promise as a tool to help people stop smoking.	79 (35.3)	91 (40.6)	46 (20.5)	8 (3.6)	0 (0.0)
There is good empirical evidence that stop smoking apps can help people quit.	53 (23.7)	100 (44.6)	59 (26.3)	11 (4.9)	1 (0.4)
As a clinician, I would recommend a stop smoking app to my patients or clients trying to quit.	85 (37.9)	74 (33.0)	56 (25.0)	6 (2.7)	3 (1.3)
Effective stop smoking apps are widely available for smokers.	55 (24.5)	57 (25.4)	91 (40.6)	19 (8.0)	2 (0.9)
If there were an app that allowed me to track my client or patients’ progress quitting smoking, I would use it as a clinician.	107 (47.7)	78 (34.8)	39 (17.4)	0 (0.0)	0 (0.0)
If there were an empirically validated stop smoking app, I would recommend it.	144 (64.3)	60 (26.8)	20 (0.1)	0 (0.0)	0 (0.0)

^a^mHealth: mobile health.

## Results

### Demographic Characteristics of Samples

A total of 319 current smokers (average cigarettes per day: mean 15, SD 12) and 224 health care providers were included in the analysis. Table 1 shows the descriptive statistics for the demographic characteristics of smokers, 92.5% (295/319) individuals were male with an average age of 34.8 years and 7.5% (24/319) were female with an average age of 23.5 years. Almost half of the smokers (148/319, 46.4%) were employed full time, whereas 12.5% (40/319) of them were students. The education level of 85.6% (273/319) of smokers was bachelor’s degree and above. The fraction of smokers from provincial capitals and higher-level cities (which includes metropolis, provincial capital, prefecture-level city, and county level [22,23]) was 63.6% (203/319). The fraction of smokers living with others was 81.2% (259/319). Only 4.4% (14/319) of the smokers had ever downloaded a smoking cessation app.

Regarding the health care providers (Table 2), 82.1% (184/224) were female, 39.7% (89/224) were aged more than 35 years, 45.1% (101/224) were nurses, and 36.2% (81/224) were physicians; the education level of 98.7% (221/224) of health care providers was bachelor’s degree or above.

### Providers and Smokers’ Attitudes and Ratings About Smoking Cessation App and Features

Providers’ attitudes and beliefs about mHealth cessation apps are summarized in Table 3. Most health care providers agreed (somewhat or completely) that mHealth apps hold promise for helping people quit smoking (170/224, 75.9%) and would recommend them to their clients (159/224, 70.9%), especially if the program was empirically validated (204/224, 91.1%). Half of the health care providers thought that effective cessation apps currently exist (112/224, 49.9%).

**Figure 1:**
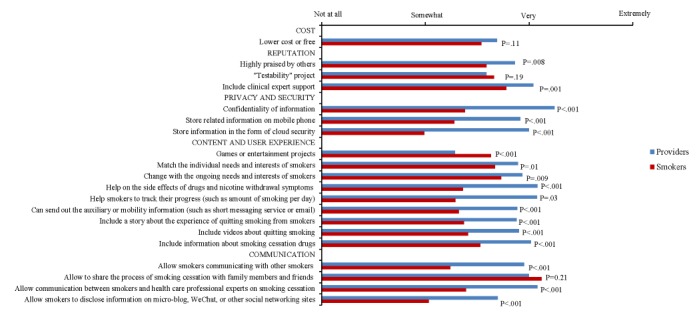
Comparison of providers’ and smokers’ ratings of important features.

Smokers’ and health care providers’ ratings of important smoking cessation app features are compared in Table 4 and Figure 1. As can been seen in Table 4, more than 60% of smokers rated the following 4 features as very or extremely important: allow sharing the process of smoking cessation with family members and friends (216/319, 67.7%), help smokers track their progress (such as amount of smoking per day; 213/319, 66.8%), help with the side effects of medications and nicotine withdrawal symptoms (201/319, 63.0%), and adapt to ongoing needs and interests of smokers (194/319, 60.8%). Almost half of the smokers (136/319, 42.6%) rated the features of *allow smokers to disclose information on microblogs, WeChat, or other social networking sites* and *include games or entertainment projects* as not at all important. In contrast, the majority of health care providers rated all the features as very or extremely important except: includes games or entertainment (91/224, 40.6%) and includes videos about quitting smoking (52/224, 23.2%).

As shown in Figure 1, health care providers rated nearly all the features as more important than the smokers did. The only 3 exceptions were the apps being lower cost or free, being research tested, and allowing sharing the process of smoking cessation with family members and friends. Among the 5 domains, smokers rated *reputation* as more important than *communication*, *privacy and security*, and *content and user experience*. Moreover, *content and user experience* and *cost* were both significantly more important than *privacy and security* to smokers. In contrast, health care providers rated the domain of *privacy and security* more important than those of *communication* and *content and user experience*. Moreover, they rated the feature of *confidentiality of information* as the most important feature for cessation apps.

Among the subgroups of smokers defined by the demographic characteristics, the reader can see in the Multimedia Appendix 1 the statistically significant differences in the 5 overall domain ratings between smokers younger than 35 years and those older than 36 years. There were no overall differences in the ratings by these subgroups we examined: occupation, education, nationality, living region, living status, tried to quit smoking in the past, used another health-related app in the past, and downloaded a smoking cessation app in the past.

**Table 4:** Smokers and health care providers’ ratings about smoking cessation apps features.

Features	Smokers, n (%)			Health care providers, n (%)		
	Not at all	Somewhat	Very or extremely	Not at all	Somewhat	Very or extremely
Allow sharing the process of smoking cessation with family members and friends	33 (10.3)	70 (21.9)	216 (67.7)	5 (2.2)	48 (21.4)	171 (76.3)
Help smokers track their progress (such as amount of smoking per day)	54 (16.9)	52 (16.3)	213 (66.8)	6 (2.7)	40 (17.9)	178 (79.5)
Help with the side effects of medications and nicotine withdrawal symptoms	58 (18.2)	60 (18.8)	201 (63.0)	7 (3.1)	42 (18.8)	175 (78.1)
Adapt to ongoing needs and interests of smokers	59 (18.5)	66 (20.7)	194 (60.8)	13 (5.8)	52 (23.2)	159 (71.0)
Keeps information private	70 (21.9)	61 (19.1)	188 (58.9)	8 (3.6)	29 (12.9)	187 (83.4)
Include clinical expert support	74 (23.2)	60 (18.8)	185 (58.0)	8 (3.6)	45 (20.1)	171 (76.4)
Match individual needs and interests of smokers	67 (21.0)	70 (21.9)	182 (57.1)	9 (4.0)	54 (24.1)	162 (71.8)
Highly rated by others	69 (21.6)	79 (24.8)	171 (53.6)	7 (3.1)	69 (30.8)	149 (66.1)
Allow communication between smokers and health care professional expert on smoking cessation	68 (21.3)	85 (26.7)	166 (52.0)	4 (1.8)	40 (17.9)	180 (80.4)
Low cost or free	72 (22.6)	90 (28.2)	157 (49.2)	15 (6.7)	81 (36.2)	128 (57.2)
Allow smokers to communicate with other smokers	83 (26.0)	89 (27.9)	147 (46.1)	6 (2.7)	53 (23.7)	165 (73.7)
Can send out auxiliary or mobility information (such as short message service or email)	76 (23.8)	99 (31.0)	144 (45.1)	8 (3.6)	64 (28.6)	152 (67.9)
Include stories about the experiences of quitting smoking from smokers	87 (27.3)	93 (29.2)	139 (43.6)	8 (3.6)	61 (27.2)	155 (69.2)
Include information about smoking cessation medications	90 (28.2)	91 (28.5)	138 (43.3)	7 (3.1)	46 (20.5)	171 (76.3)
Stores information on phone	77 (24.1)	107 (33.5)	135 (42.3)	12 (5.4)	57 (25.4)	155 (69.2)
Stores information in secure cloud	96 (30.1)	98 (30.7)	125 (39.2)	12 (5.4)	50 (22.3)	162 (72.3)
Program is *research tested*	96 (30.1)	104 (32.6)	119 (37.3)	16 (7.1)	91 (40.6)	117 (52.2)
Include videos about quitting smoking	108 (33.9)	99 (31.0)	112 (35.1)	7 (3.1)	60 (26.8)	157 (23.2)
Allow smokers to disclose information on microblogs, WeChat, or other social networking sites	136 (42.6)	87 (27.3)	96 (30.1)	24 (10.7)	68 (30.4)	132 (58.9)
Include games or entertainment	136 (42.6)	88 (27.6)	95 (29.8)	58 (25.9)	75 (33.5)	91 (40.6)

## Discussion

### Principal Findings

As far as we know, this was the first study to assess Chinese providers’ and smokers’ preferences for features of smoking cessation apps. The results offer researchers and developers guidance about what features might increase engagement with future smoking cessation apps [8]. Smokers rated *reputation* as more important than *communication*, *privacy and security*, and *content and user experience*. In traditional Chinese culture, the choice of products or services comes largely from the recommendation of friends or experts [23,24]. They are often unwilling to try new services without a reference from a trusted source [25]. Therefore, source expertise (eg, includes clinical expert support) and source trustworthiness (eg, highly praised by others and *research tested*) were rated very high among features of smoking cessation apps. Moreover, *content and user experience* and *cost* were both significantly more important than *privacy and security* to smokers.

In contrast, providers rated the domain of *privacy and security* more important than those of *communication* and *content and user experience*. Moreover, they rated the feature of *confidentiality of information* as the most important feature for cessation apps, which is consistent with the opinions of American providers [16]. The importance of privacy and security likely reflects the obligation of providers to protect the privacy of patients. Furthermore, providers rated the ability of an app to help smokers track their progress as well as obtain information about smoking cessation medications, side effects of nicotine withdrawal symptoms, and support and communication with clinical expertise as very important. As most smoking cessation apps in the Chinese market do not currently provide these functionalities [10], it would seem important to add them in future apps. In contrast with American providers [16], Chinese providers rated games or entertainment projects as the least important.

In the content and user experience domain, having the app change to meet ongoing needs and interests was rated highly both by smokers and providers. This suggests that future smoking cessation apps should design content to include adaptively tailored features (eg, weekly graph based on the personal data smokers enter). The value of adaptive content is supported by a variety of behavior change theories [26,27].

Providers were generally supportive of apps for smoking cessation. The majority stated that they would recommend them and they would use them to track their patients’ progress. Interestingly, a substantial fraction (49.9%) of health care providers thought that effective cessation apps currently exist, a belief that is not supported by the nascent research on these apps [28]. This may mean that this misperception is leading providers to incorrectly recommend apps for which there is no empirical support. Future app designs should be very clear about the current evidence to date on their effectiveness.

Comparing the results of this study of Chinese smokers with the previous study of US smokers reveals some intriguing contrasts. Specifically, security was rated as the least important domain by Chinese smokers in our study, whereas smokers in the US study rated that as the most important feature [16]. This contrast may be because smoking is not a private behavior in China: it functions as a way to make social connections, make smooth social interactions, and express one’s social and economic position [29]. In contrast, smoking in America is highly stigmatized, which can have iatrogenic effects on quitting smoking [30]. Moreover, studies showed that Chinese people are more open and less sensitive about privacy [31]. According to a report published by market research firms (Experian and International Data Corporation) [32], people’s attitudes toward Web-based privacy vary widely from country to country, with Chinese people being among the most willing to sacrifice privacy for safety and convenience. Therefore, privacy in quitting smoking would be highly valued for American smokers but not for Chinese smokers. Moreover, a second contrast with the US study was that Chinese smokers and providers of this study rated *allowing sharing of the process of smoking cessation with family members and friends* (communication domain) as the most important feature (*P*>.05), whereas the American smokers and providers rated that as relatively unimportant. This contrast likely reflects differences in Chinese values of family and close relationships, with behavior change involving the collective input of those people. Loved ones are seen as key sources of support to change a health habit in China [33].

### Design Recommendations

The results yield a number of recommendations for the design of a smoking cessation app for Chinese smokers. Features rated very highly by both the smoker and provider samples suggest that an app should focus on (1) facilitating support for cessation from family and friends, (2) tracking their progress with quitting smoking, (3) getting help with the side effects of medications and nicotine withdrawal, and (4) being tailored to their unique needs. Results from the provider sample also suggest that an app should focus on protecting the privacy and confidentiality of the user as such a feature may make it more likely for providers to recommend the app to their patients. At the same time, these features rated consistently lower by both the smoker and provider samples suggest that an app for Chinese smokers should not (1) include games or entertainment and (2) allow disclosure on microblogs or WeChat.

### Strengths and Limitations

This study has some notable strengths. This was the first study to investigate the opinions of Chinese health care providers and smokers on features in a quit smoking smartphone app. This study has limitations that can be addressed in future studies. First, as there is a broad diversity of smokers and providers in China, it is not possible to know to what extent the current sample’s results can be generalized to the broader population of Chinese smokers or providers. Second, what smokers say they prefer may be different from what they actually will find useful once an app is prototyped. Thus, this study needs to be viewed as an important starting point in the design process. In-depth qualitative interviews and presenting early design sketches to smokers and providers would be the natural next steps in the process of creating a specific app.

### Conclusions

Chinese health care providers expressed strong support of smoking cessation apps and indicated that nearly all features were important. Contrary to a similar study of US smokers and health care providers, Chinese smokers and providers highly value a smoking cessation app’s reputation and ability to communicate with family members and friends as important features, whereas Chinese smokers rated privacy and security as less important. Therefore, the design of future smoking cessation and health behavior change apps should consider cultural differences and the perspectives of both health care providers and smokers.

## Appendix

Multimedia Appendix 1 Ratings of features among subgroups with different ages (%).

## Abbreviations

mHealth: mobile health
