# Supplementary material for: Design and Development of Smoking Cessation Apps Based on Smokers’ and Providers’ Perspectives in China: Survey Study
Source: JMIR Mhealth Uhealth. 2019 Oct 4;7(10):e12200. doi: 10.2196/12200 (PMC6800458; doi:10.2196/12200)
Supplement: Multimedia Appendix 1 [file mhealth_v7i10e12200_app1.pdf]

Supplementary Table Ratings of features among subgroups with different ages. (%)

	≤35			≥36			P
Items	Not at all	Somewhat	Very or extremely	Not at all	Somewhat	Very or extremely	
Allow sharing the process of smoking cessation with family members and friends	27.59	21.84	50.57	24.82	31.21	43.97	0.573
Help smokers track their progress(such as amount of smoking per day)	13.22	14.37	72.41	20.57	18.44	60.99	0.028
Help with the side effects of medications and nicotine withdrawal symptoms	16.09	18.39	65.52	19.86	19.86	60.28	0.311
Adapt to ongoing needs and interests of smokers	15.52	19.54	64.94	21.28	22.70	56.03	0.094
Confidentiality of information	20.69	14.94	64.37	22.70	24.82	52.48	0.079

Include clinical expert support	18.97	17.24	63.79	27.66	21.28	51.06	0.020
Match individual needs and interests of smokers	18.39	22.41	59.20	23.40	21.99	54.61	0.321
Highly praised by others	17.82	19.54	62.64	25.53	31.91	42.55	0.001
Allow communication between smokers and healthcare professional experts on smoking cessation	21.84	20.11	58.05	19.86	34.75	45.39	0.129
Low cost or free	18.97	28.74	52.30	26.24	28.37	45.39	0.133
Allow smokers to communicate with other smokers	22.99	22.41	54.60	29.08	34.75	36.17	0.005
Can send out auxiliary or mobility information(such as SMS or email)	21.84	31.61	46.55	25.53	30.50	43.97	0.512
Include stories about the experiences of quitting smoking from smokers	25.29	28.74	45.98	29.08	29.79	41.13	0.354
Include information about smoking cessation medications	27.01	25.29	47.70	29.08	32.62	38.30	0.194

Store related information on mobile phone	19.54	32.18	48.28	29.08	36.17	34.75	0.010
Store information in the form of Cloud Security	29.31	28.74	41.95	30.50	34.04	35.46	0.392
“Testability” project	27.01	28.74	44.25	33.33	38.30	28.37	0.014
Include videos about quitting smoking	33.33	27.01	39.66	33.33	36.88	29.79	0.287
Allow smokers to disclose information on microblogs, WeChat, or other social networking sites	39.66	21.84	38.51	45.39	34.75	19.86	0.016
Include games or entertainment projects	41.95	22.99	35.06	42.55	34.04	23.40	0.244
